# Prevalence and Impact of Minority Variant Drug Resistance Mutations in Primary HIV-1 Infection

**DOI:** 10.1371/journal.pone.0028952

**Published:** 2011-12-16

**Authors:** Joanne D. Stekler, Giovanina M. Ellis, Jacquelyn Carlsson, Braiden Eilers, Sarah Holte, Janine Maenza, Claire E. Stevens, Ann C. Collier, Lisa M. Frenkel

**Affiliations:** 1 Department of Medicine, University of Washington, Seattle, Washington, United States of America; 2 Department of Laboratory Medicine, University of Washington, Seattle, Washington, United States of America; 3 Center for AIDS and STDs, University of Washington, Seattle, Washington, United States of America; 4 Seattle Children's Hospital and Research Institute, Seattle, Washington, United States of America; 5 Fred Hutchinson Cancer Research Center, Seattle, Washington, United States of America; University of California San Francisco, United States of America

## Abstract

**Objective:**

To evaluate minority variant drug resistance mutations detected by the oligonucleotide ligation assay (OLA) but not consensus sequencing among subjects with primary HIV-1 infection.

**Design/Methods:**

Observational, longitudinal cohort study. Consensus sequencing and OLA were performed on the first available specimens from 99 subjects enrolled after 1996. Survival analyses, adjusted for HIV-1 RNA levels at the start of antiretroviral (ARV) therapy, evaluated the time to virologic suppression (HIV-1 RNA<50 copies/mL) among subjects with minority variants conferring intermediate or high-level resistance.

**Results:**

Consensus sequencing and OLA detected resistance mutations in 5% and 27% of subjects, respectively, in specimens obtained a median of 30 days after infection. Median time to virologic suppression was 110 (IQR 62–147) days for 63 treated subjects without detectable mutations, 84 (IQR 56–109) days for ten subjects with minority variant mutations treated with ≥3 active ARVs, and 104 (IQR 60–162) days for nine subjects with minority variant mutations treated with <3 active ARVs (p = .9). Compared to subjects without mutations, time to virologic suppression was similar for subjects with minority variant mutations treated with ≥3 active ARVs (aHR 1.2, 95% CI 0.6–2.4, p = .6) and subjects with minority variant mutations treated with <3 active ARVs (aHR 1.0, 95% CI 0.4–2.4, p = .9). Two subjects with drug resistance and two subjects without detectable resistance experienced virologic failure.

**Conclusions:**

Consensus sequencing significantly underestimated the prevalence of drug resistance mutations in ARV-naïve subjects with primary HIV-1 infection. Minority variants were not associated with impaired ARV response, possibly due to the small sample size. It is also possible that, with highly-potent ARVs, minority variant mutations may be relevant only at certain critical codons.

## Introduction

Transmission of drug resistant HIV-1 has been well-documented following the widespread availability of antiretroviral (ARV) therapy [1]–[11]. In the United States, cross-sectional surveys using consensus sequencing estimate that 11–24% of persons acquire drug resistant HIV-1 [8], [9], [12], [13]. National guidelines therefore recommend genotypic resistance testing for ARV-naïve persons at entry into care [14].

Consensus sequencing cannot consistently detect viral variants unless they comprise greater than 10–50% of the population [15]–[20], and studies using more-sensitive assays have detected mutations at lower concentrations (i.e. “minority variant” mutations) in up to half of ARV-naïve subjects [21]–[24]. However, the impact of minority variants on HIV-1 disease progression and response to ARVs remains unclear. Some studies have found associations between minority variant mutations and poor clinical outcomes [23]–[30] or that outcomes were dependent on the specific mutant codon and ARV therapy used [23], [30], [31], but others have found no association between minority variants and treatment responses [32]–[34].

The oligonucleotide ligation assay (OLA) is an HIV-1 drug resistance assay that is more sensitive than consensus sequencing and can detect mutations at select codons when they occur in as few as 2–5% of the viral quasi-species [35]–[38]. This study evaluated the prevalence of mutations detected by OLA and the impact of minority variants on responses to ARV therapy in a cohort of subjects with primary HIV-1 infection.

## Materials and Methods

### Patient population

Characteristics of the University of Washington Primary Infection Clinic (PIC) cohort have previously been described [39]–[41]. For this project, we selected a subgroup from among 201 subjects in the cohort who acquired HIV-1 after highly active ARV therapy became widely available in 1996. We preferentially selected subjects who 1) had enrolled in the cohort within one month of their estimated date of HIV-1 infection (defined as the date of onset of seroconversion symptoms or, for asymptomatic individuals, the midpoint between dates of the last negative and first positive HIV-1 tests), 2) had results of a pre-treatment HIV-1 drug resistance test (consensus sequencing) already available, and/or 3) initiated ARV therapy within six months of study enrollment. We performed consensus sequencing and sensitive drug resistance testing to determine HIV-1 genotype on the first available (i.e. baseline) plasma and peripheral blood mononuclear cell (PBMC) specimens that had been collected no more than seven days after the start of ARVs. Thirty-four subjects had consensus sequencing performed as part of clinical research evaluations prior to our undertaking this analysis; results of resistance testing performed specifically for this study were not used to guide selection of ARV therapy. This study was approved by the University of Washington Institutional Review Board, and all subjects gave written consent for participation in the cohort.

### HIV-1 RNA quantification in blood plasma

Specimens collected between 1996 and 2002 were initially tested with branched DNA (bDNA) assays with lower limits of detection of 50 and 500 copies/mL (Chiron Corporation; Emeryville, CA). When specimens were available, results censored at 500 copies/mL were re-tested using an ultra-sensitive reverse transcription polymerase chain reaction (RT-PCR) assay (Roche; Branchburg, NJ) or an independently-validated real-time RT-PCR amplification assay with lower limits of detection equal to 50 copies/mL [42]. Since 2002, all specimens have been evaluated by an RT-PCR assay.

### RT-PCR and PCR for genotyping of HIV-1 *pol*

RNA was extracted from plasma and reverse transcribed, as previously described [35]. DNA was extracted from PBMCs using the Puregene Cell and Tissue kit (Gentra Systems, Inc.; Minneapolis, MN) according to manufacturers' instructions. Nested PCR was performed as previously described [37] with different primers. Briefly, first-round PCR of cDNA or DNA was carried out in a 50-µl reaction mixture containing 10-µl of cDNA or ≥1 µg DNA, and second-round PCR contained 2-µl of the first-round product. First round primers were PRA and RTA; second round primers were PRB and RT3 [43]. If no amplicon was produced, we used alternate primer sets NE10 and NE11. We visualized the amplicon, a 1,193-bp DNA fragment extending from amino acid 1 in protease to amino acid 230 in reverse transcriptase, in a 1% agarose gel with ethidium bromide staining. Samples with a visible band of the correct size were used for sequencing and OLA.

### Consensus sequencing

PCR amplicons were purified and sequenced as previously described [37] using sequencing primers that were identical to those used for second-round PCR. Sequences were analyzed with Sequencher, version 4.2 (Gene Codes Corp; Ann Arbor, MI), and submitted to the Stanford HIV-1 Sequence Analysis Program [44] to identify mutations. For quality assurance, all genotypes generated for this study were aligned with ClustalW, v1.81 and reviewed using a neighbor-joining phylogenetic tree to monitor for cross-contamination.

### Oligonucleotide Ligation Assay (OLA)

Amplicons submitted to consensus sequencing were evaluated by OLA for mutations in the region encoding reverse transcriptase (K65R, K70R, L74V, M184V, T215F/Y, K103N, Y181C, and G190A) and protease (D30N, I50V, V82S/A/T, I84V, N88D, and L90M). Results for M41L are not included, as oligonucleotide probes for this codon were not optimized when the laboratory work for this project was completed. OLA was performed as previously described [35]–[37]. All subjects' samples and controls were analyzed in duplicate. We classified samples as mutant if the mean optical density (OD) of duplicates at 490 nm was greater than the OD of the 5% mutant control or 2.5 times the OD of the wild-type control. If the specimen was not classified as mutant and the mean wild-type OD was under 50% of the wild-type control OD, we classified the specimen as “indeterminate.”

### Statistical analysis

We used McNemar's exact tests to compare the number of subjects with transmitted drug resistance mutations detected by OLA and consensus sequencing of plasma, by OLA and consensus sequencing of PBMCs, and by OLA of plasma to OLA of PBMCs. Multivariable regression models explored factors potentially associated with risk of transmitted drug resistance and included year of HIV-1 acquisition (divided into quartiles) to evaluate for evidence of a secular trend.

We used 2-sample t-tests, non-parametric tests, and regression analyses where appropriate to compare mean baseline (i.e. first visit) CD4^+^ T-cell count, baseline HIV-1 RNA level, and median viral “set point” among subjects with minority variant mutations and subjects without detectable transmitted drug resistance. We estimated set point using the HIV-1 RNA level obtained closest to 150 days (between 120 and 730 days) following HIV-1 infection from subjects who had not yet received ARV therapy [45].

We conducted time-to-event analyses using Cox proportional hazard regression models with maximum likelihood estimation to compare time to virologic suppression (defined as the first HIV-1 RNA below 50 copies/mL) among subjects receiving highly active antiretroviral therapy. These analyses were adjusted for pre-treatment HIV-1 RNA level closest to and within 30 days before the start of ARVs. We excluded subjects from the time-to-event analyses if they received only single or dual nucleoside reverse transcriptase inhibitor (NRTI) therapy or if they had any mutations detected by consensus sequencing because pre-treatment resistance testing could have guided selection of ARVs. The Stanford University HIV Drug Resistance Database (http://hivdb.stanford.edu; accessed December 29, 2009) was used to predict the number of active agents in regimens; an ARV agent was considered inactive if subjects had a mutation associated with intermediate or high-level HIV-1 drug resistance to that ARV. Subjects were divided into three groups: 1) subjects without any HIV-1 drug resistance mutations detected by consensus sequencing or OLA, 2) subjects with minority variant mutations treated with ARV regimens with three or more active ARV agents, and 3) subjects with minority variant mutations treated with ARV regimens with fewer than three active agents. Virologic failure was defined as: 1) failure to suppress HIV-1 RNA levels to below 50 copies/mL within 240 days after initiation of ARVs, 2) switch of ARV agents due to a perceived inadequate response to therapy, or 3) viral rebound to greater than 500 copies/mL on two consecutive measurements following successful suppression of HIV-1 RNA levels to below 50 copies/mL. All statistical analyses were performed using Stata9 software (StataCorp LP, College Station, TX).

## Results

Demographics and other baseline characteristics of the 99 subjects are shown in **Table 1**. All subjects were men, and 98% of subjects reported sex with men as their risk for HIV-1 acquisition. Subjects who experienced symptoms consistent with the acute retroviral syndrome (92%) were over-represented in this analysis compared to the entire PIC cohort (84%). All subjects acquired HIV-1 subtype B infection.

**Table 1:** Characteristics of study subjects with primary HIV-1 infection evaluated for drug resistance mutations.

		No mutations	Mutations by OLA only	Mutations by CS	All Subjects	
		n = 72	n = 22	n = 5	n = 99	
Age (median, IQR)		34 (30–38)	37 (30–43)	33 (33–41)	34 (30–40)	NS
Caucasian, non-Hispanic		92%	95%	100%	93%	NS
Days from infection to screening (median, IQR)		22 (13–52)	32 (24–63)	54 (34–80)	27 (15–63)	p = .05
CD4^+^ T-cell count at first visit (median cells/mm^3^, IQR)		494 (386–678)	551 (394–700)	421 (396–550)	496 (392–694)	NS*
HIV RNA level at first visit (median log_10_ copies/mL, IQR)		5.4 (4.5–6.2)	5.1 (4.6–5.5)	5.2 (5.0–6.0)	5.2 (4.5–6.0)	NS*
Median date of HIV infection (IQR)		8/01 (1/00-1/04)	4/02 (4/01-1/04)	1/02 (8/00-4/04)	11/01 (4/00-2/04)	NS
Received ARV treatment		64 (89%)	20 (91%)	5 (100%)	89 (90%)	NS
	PI/NRTI	26 (41%)	7 (35%)	2 (40%)	35 (39%)	NS
Initial	NNRTI /NRTI	24 (38%)	9 (45%)	2 (40%)	35 (39%)	
ARV	PI/NNRTI/NRTI	13 (20%)	3 (15%)	1 (20%)	17 (19%)	
regimen	NRTI only	1 (2%)	1 (5%)	0	2 (2%)	

OLA: oligonucleotide ligation assay; CS: consensus sequencing; IQR: interquartile range; NS: not significant at p = .05; ARV: antiretroviral; PI: protease inhibitor; NRTI: nucleoside reverse transcriptase inhibitor; NNRTI: non-nucleoside reverse transcriptase inhibitor therapy.

*differences between groups were not significant in analyses that were both unadjusted and adjusted for time from infection to the date of sampling.

We performed HIV-1 drug resistance testing on plasma and PBMC specimens that had been obtained a median of 29 (IQR 19–66) and 31 (IQR 19–66) days after HIV-1 infection; all specimens were collected within six months of infection. Consensus sequencing and OLA detected HIV-1 drug resistance mutations (in either plasma or PBMCs) in 5% and 27% of 99 subjects, respectively. There was no evidence of a trend in incidence of transmitted drug resistance over time, although resistance was more common among subjects infected after May 2000 (31%) compared to those infected prior to this date (16%), and we did not detect non-nucleoside reverse transcriptase inhibitor (NNRTI) resistance among subjects infected prior to this date.

Compared to consensus sequencing, OLA detected significantly greater number of subjects with resistance mutations in both plasma (p = .0005) and PBMCs (p = .002) (**Table 2**). OLA performed on plasma and PBMCs detected similar numbers of subjects with drug resistance mutations, but concordance of results was low.

**Table 2:** HIV-1 drug resistance in ARV-naïve subjects with primary HIV-1 infection.

2a: OLA versus consensus sequencing of plasma				2b: OLA versus consensus sequencing of PBMC				2c: Consensus sequencing of plasma versus PBMC				2d: OLA of plasma versus PBMC			
p = .0005		OLA		p = .002		OLA		p = 1.0		PBMCs		p = 1.0		PBMCs	
		−	+			−	+			−	+			−	+
sequencing	−	83	**12**	sequencing	−	81	**13**	plasma	−	94	**1**	plasma	−	72	**11**
	+	**0**	4		+	**1**	4		+	**0**	4		+	**10**	6

ARV: antiretroviral; OLA: oligonucleotide ligation assay; PBMC: peripheral blood mononuclear cells.

+ = subjects with ≥1 mutation or mixture.

− = subjects without mutations or with indeterminate results.

Numbers represent subjects in whom HIV-1 drug resistance was/was not detected in plasma and PBMC specimens that had been obtained a median of 29 (IQR 19–66) and 31 (IQR 19–66) days after HIV-1 infection, respectively; all specimens were collected within six months of infection. McNemar's exact tests compare only subjects with discordant results (indicated in bold).

Consensus sequencing detected one subject with M184V in PBMCs only, one subject with M41L and T215D, one subject with T215D and L90M, and two subjects with G190A. The mutations most commonly identified by OLA in reverse transcriptase were M184V (n = 9) and T215Y (n = 5) and in protease were I84V (n = 5) and N88D (n = 5). K103N was identified in only one subject and by OLA only. With use of OLA, detection of NRTI resistance mutations increased from 3% to 13% of subjects, detection of NNRTI resistance mutations increased from 2% to 8% of subjects, detection of protease inhibitor (PI) resistance mutations increased from 1% to 13% of subjects, and detection of multi-drug resistant HIV-1 increased from 1% to 6% of subjects.

Compared to subjects without detectable mutations, there were no differences in the CD4^+^ T-cell counts or HIV-1 RNA levels at presentation among subjects having at least one mutation detected by OLA or subjects with mutations conferring resistance to NRTIs, NNRTIs, or PIs. Among 24 subjects in this study who remained untreated at a median of 146 days following HIV-1 infection, the median viral “set point” was 4.5 (IQR 3.7–4.8) log_10_ copies/mL among five subjects with minority variant mutations and 4.5 (IQR 4.1–5.3) log_10_ copies/mL among 19 subjects with no detectable drug resistance mutations (p = .6).

Eighty-nine (90%) of the 99 subjects initiated ARV therapy a median of 48 (IQR 24–107, range 5–1092) days after HIV-1 infection (**Table 3**). Mean CD4^+^ T-cell counts (498 versus 486 cells/mm^3^, p = .8) and HIV-1 RNA levels (5.1 versus 4.9 log_10_ copies/mL, p = .5) at the start of ARV therapy did not differ between subjects without detectable mutations and those with minority variant mutations. Similarly, we found no association between any class of drug resistance mutation and CD4 count or HIV-1 RNA level at the start of ARV therapy.

**Table 3:** HIV-1 drug resistance detected by consensus sequencing and OLA in ARV-naïve subjects and virologic response to ARV therapy.

Group	ID	Infection Year	CS (plasma)	CS (PBMC)	OLA (plasma)	OLA (PBMC)	VL at ARV start^1^	Initial ARVs	#Active ARVs	Time to VL<50 (days)	VF
	95816	2000	G190A	G190A	G190A	G190A	4.9	ABC, 3TC, EFV, RTV-APV→ABC, 3TC, NVP	4→2^2^	57	yes
	69234	2004	G190A	G190A	G190A	M184V, T215Y, G190A	5.6	AZT, 3TC, r-LPV	1	87^3^	
**I**	43909	2002		M184V		M184V	4	ABC, 3TC, EFV	2	27	
	56710	1999	M41L, T215D	M41L, T215D	T215Y^4^	4 ^,^ 5	4.9	3TC, d4T, IDV, HU	2	176	
	35188	2004	T215D, L90M	T215D, L90M	L90M^4^	L90M^4^	5.4	TDF, FTC, EFV	3	216	
	34993	2000			V82A		4.1	ABC, 3TC, IDV, EFV	3	15	
	41319	2002			I84V, N88D		4.2	ABC, 3TC, EFV	3	55	
	19198	2003			Y181C, I84V		5.4	AZT, 3TC, r-LPV	3	56	
	83622	2000				M184V	5.3	ABC, 3TC, IDV, EFV	3	60	
**II**	35057	2004				I84V	5	ABC, 3TC, EFV	3	84	
	18309	2003			N88D	D30N	5.4	ABC, 3TC, EFV	3	85	
	44375	2003				N88D	4.5	ABC, 3TC, r-IDV	3	104	
	66121	2001			150V	N88D	4.5	ABC, 3TC, EFV	3	109	
	28477	2005				K103N	4.3	TDF, FTC, r-LPV	3	181	
	50047	2004				I84V	4.8	ABC, 3TC, EFV	3	266	
	78056	2003			I84V		4.1	ABC, 3TC, r-IDV	2	55	
	97929	2001				M184V	4.5	ABC, 3TC, EFV	2	60	
	71670	2000			M184V, Y181C		4.5	ABC, 3TC, IDV, EFV	2	84	
	81563	2001				M184V	4.9	ABC, 3TC, r-IDV	2	104	
**III**	53754	2000				M184V, N88D	4.8	3TC, d4T, NVP	2	162	
	49635	2001			M184V, Y181C		4.7	ABC, 3TC, r-IDV	2	165	
	26973	2002				T215Y	5	ABC, AZT, 3TC	2	DNS^6^	yes
	44378	2005			Y181C		5.9	AZT, 3TC, NVP	2	DNS^6^	
	78882	2004			K65R, M184V, T215Y, I50V		5.3	TDF, FTC, r-ATZ	1	DNS^6^	

In this table, the subset of subjects who received antiretroviral (ARV) therapy are grouped based on whether they had drug resistance detected by consensus sequencing (Group I), drug resistance detected by OLA but who received at least three active ARV agents (Group II), or drug resistance detected by OLA who received fewer than three active agents (Group III). ARVs are highlighted in grey if subjects had mutations conferring at least intermediate level resistance to that ARV. K70R, L74V, T215F, and V82S/T were not detected in any treated subjects.

CS: consensus sequencing; OLA: oligonucleotide ligation assay; VL: viral load (HIV-1 RNA level); ARV: antiretroviral; VF: virologic failure; IDV: indinavir, HU: hydroxyurea, ABC: abacavir, EFV: efavirenz, NVP: nevirapine, r-: ritonavir-boosted, LPV: lopinavir, ATZ: atazanavir; DNS: did not suppress.

1 Log_10_ copies/mL.

2 Antiretroviral medications were switched on day 5 due to side effects.

3 Subject #69234 subsequently discontinued medications two months later due to adherence difficulties.

4 OLA probes did not test for M41L and T215D.

5 OLA on PBMC for subject 56710 yielded indeterminate results for T215Y.

6 DNS: did not suppress prior to discontinuing ARVs or study censorship. Subjects #26973, 44378, and 78882 were followed for 104, 44, and 63 days, respectively, while receiving ARVs.

**Figure 1:**
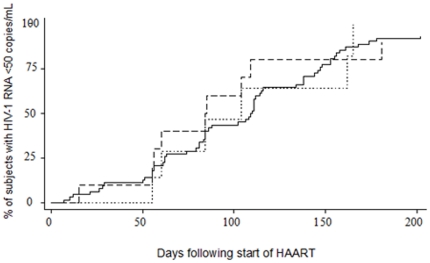
Time to suppression of plasma HIV-1 RNA levels among previously ARV-naïve subjects with and without minority variant drug resistance mutations. Figure 1: The median time to virologic suppression (HIV-1 RNA<50 copies/mL) was 110 (IQR 62–147) days for 63 treated subjects without detectable mutations (solid line), 84 (IQR 56–109) days for 10 subjects with minority variant mutations treated with ≥3 active ARVs (dashed line), and 104 (60–162) days for nine subjects with minority variant mutations treated with <3 active ARVs (dotted line) (p = .9).

The five subjects with mutations detected by consensus sequencing and two other treated subjects without follow-up were excluded from survival analyses. Median time to HIV-1 RNA less than 50 copies/mL was 110 (IQR 62–147) days for 63 treated subjects without detectable mutations, 84 (IQR 56–109) days for ten subjects with minority variant mutations treated with three or more active ARVs, and 104 (IQR 60–162) days for nine subjects with minority variant mutations treated with fewer than three active ARVs (p = .9) (**Figure 1**). After adjustment for HIV-1 RNA levels at the start of ARV therapy, time to virologic suppression was similar (aHR 1.2, 95% CI 0.6–2.4, p = .6) for subjects with minority variant mutations treated with at least three active agents and for subjects with minority variant drug resistance mutations who received fewer than three active ARV agents (aHR 1.0, 95% CI 0.4–2.4, p = .9) compared to subjects without drug resistance mutations.

The eighty-seven treated subjects, including the five subjects with mutations identified by consensus sequencing, were followed for a median of 4.4 (IQR 2.6–7.7) person-years following the start of ARV therapy. Only four (5%) subjects experienced virologic failure. One subject (**Table 3** ID #95816) did not have resistance testing performed prior to starting ARV therapy. After virologic failure, G190A was identified in his baseline specimen by both consensus sequencing and OLA. The second subject with virologic failure (ID #26973) had no major mutations identified by consensus sequencing. His HIV-1 RNA level decreased to 2.0 log_10_ copies/mL before it quickly rebounded; T215Y was detected by only OLA in his PBMCs from baseline. Drug resistance mutations were not identified at baseline in two other subjects who experienced virologic failure after receiving ARV therapy for five months and five years;

## Discussion

The results described here represent one of the most comprehensive surveys of minority variant drug resistance in primary HIV-1 infection in terms of the number of mutations studied. We detected drug resistance mutations in 27% of a male cohort who acquired HIV-1 infection after 1996. Despite a high prevalence of minority variant drug resistance mutations, this was not associated with a difference in viral set point or in the virologic response to ARV therapy among treated subjects.

The finding that a sensitive assay detected HIV-1 drug resistance mutations in a greater number of subjects compared to consensus sequencing is consistent with other studies of ARV-naïve subjects with primary [21], [32] and established HIV-1 infection [24], [46]. Although the high prevalence of minority variants in our subjects is somewhat incongruous with the previously-held belief that sexual transmission of HIV infection is predominantly monophyletic, more recent data have suggested that men who have sex with men frequently acquire multiple variants [47]. It is also possible that OLA detected variants that had been spontaneously generated by random misincorporation of base pairs during reverse transcription of HIV RNA. However, although possible, it is unlikely that, without ARV selection pressure, mutations could be generated with sufficient frequency to reach a level detectable by OLA in nearly one quarter of our subjects [48]. It is also conceivable that false positive results contributed to the estimated prevalence of drug resistance in our subjects. Although ligase binding is highly specific [49], false positive results could have occurred due to high background in the EIA portion of the assay for isolated specimens.

In contrast to our previous study of ARV-experienced persons with chronic HIV-1 infection [37], OLA of PBMC DNA did not detect a greater number of persons with mutations compared to OLA of plasma RNA. All minority variant drug resistance mutations detected in this study were identified in only one component of blood (i.e. either plasma or PBMCs but not both). These specimens had concentrations of mutant virus that were close to the limit of detection of the assay, and thus detection in plasma or PBMCs was likely stochastic. We suspect that the reason the numbers of persons who had mutations detected in one component or the other were similar was due to collection of specimens during primary infection with insufficient time lapse for wild-type viruses to have overgrown less fit mutants in plasma, where virus turnover occurs more rapidly. One of the strengths of this work is that we studied subjects close to the time of HIV acquisition, as outgrowth of some wild-type viruses can occur very quickly [50], [51]. We also cannot exclude the possibility that minority variants were spontaneously generated, as mentioned above.

Similar to other studies [32]–[34], we found that low-level mutations did not appear to affect the time to virologic suppression following initiation of ARV therapy in treated subjects. In contrast, studies of persons with established HIV-1 infection have shown an increased risk of virologic failure associated with minority variant drug resistance mutations [23], [24], [26], [27], [29], [30], particularly with NNRTI mutations [28]–[31]. The high rate of treatment success among our subjects was similar to another observational study of subjects with primary HIV-1 infection [52], but as a result only 4% of subjects were observed to have virologic failure and our study was underpowered to detect differences in clinical outcomes. Although the median follow-up time in our study was longer than other studies that observed higher rates of virologic failure, it is possible we would have seen differences in rates of virologic failure if subjects had remained on treatment and in follow-up or if we had studied more subjects.

Another likely explanation for our failure to identify negative consequences from minority variant mutations was the lack of uniformity of the impact of HIV-1 drug resistance mutations across regimens and the use of ARV therapy with a high genetic barrier to resistance to the mutations we observed. Much prior research on this topic has focused on NNRTI mutations, which have a lower genetic barrier to resistance. Of the two subjects in our cohort with NNRTI mutations who were treated with NNRTI-based regimens, the one who had high concentrations of mutant experienced virologic failure (ID#95816). The second subject (ID#44378) had the Y181C mutation detected only by OLA; he was treated with nevirapine and had an initial >3 log_10_ copies/mL decrease in his HIV-1 RNA level, but he discontinued medications after forty-four days due to rash.

It is possible that the clinical impact of minority variant drug resistance mutations may be modified by the relative concentration of the mutant virus at specific codons [23], [30], [53]. In one recent study, subjects who had NNRTI resistance mutations detected in 1–20% of the viral population had a lower risk of virologic failure following initiation of ARV therapy compared to subjects who had NNRTI resistance mutations detected in greater than 20% of the population, but both groups had a greater risk of virologic failure compared to subjects who did not have NNRTI resistance mutations [23]. These authors did not observe a similar relationship between risk of virologic failure and variation in concentrations of NRTI or PI mutations. Another recent study suggested that the K103N mutation was associated with an increased risk of virologic failure when these viruses were present in amounts greater than 2000 copies/mL [54]. However, like many previously published studies, we did not quantify the amount of virus used for drug resistance assays and therefore cannot report the precise concentrations of minority variants that were detected.

It is also possible that the clinical impact of minority variant transmitted drug resistance mutations may be further modified by the persistence or decay in concentration of the mutant virus. In several studies, receipt of single dose nevirapine (SD-NVP) has been associated with poor subsequent response to NVP-based ARV therapy when treatment was initiated within six months of the SD-NVP [55], [56]. It is plausible that the interaction between delayed initiation of ARV therapy following SD-NVP and reduced risk of virologic failure is mediated by decay in the concentration of HIV-1 drug resistant variants over time. In the study by Jourdain et al. [56], risk for virologic failure was associated with detection of mutations by OLA at the time of initiation of ARVs but not with detection of mutations by consensus sequencing ten days post-partum. How the specific mutations, threshold levels, dynamics of decay, and timing and type of ARV therapy all interact to modify the effect of transmitted drug resistance remains to be determined. A longitudinal study is ongoing that will quantify minority variants over time in a greater number of subjects with primary HIV-1 infection.

Although OLA is more sensitive than consensus sequencing, more sensitive assays such as allele-specific PCR [57], [58] and parallel allele-specific sequencing (PASS) [59] can detect HIV-1 drug resistance mutations in as little as 0.01–1% of the viral population if sufficient numbers of viruses are studied. Had we used one of these assays, it is possible that we would have estimated that the prevalence of HIV-1 drug resistance was even greater, and we might have found a relationship between drug resistance and virologic response to therapy. However, in our hands, OLA and pyrosequencing produce similar results when mutant viruses are at concentrations greater than 2% of the viral population [60], and the clinical relevance of minority variants at even lower concentrations is even less clear. At the highest sensitivity, it is possible to detect and misclassify random mutations, as some mutations were detected among HIV-infected persons even prior to the availability of ARVs [48], [61]. Advantages of OLA include the greater specificity compared to PCR-based methods [62], reagents anneal at relatively low temperature (37°C) and therefore tolerate polymorphisms in the region of the probe, OLA uses relatively less costly equipment than allele-specific PCR and pyrosequencing, and oligonucleotides have been adapted to non-B subtypes [63].

In conclusion, results from this study reinforce findings of others that consensus sequencing significantly underestimates the point prevalence and possibly the “persistence” of transmitted HIV-1 drug resistance mutations. However, additional data are still needed to precisely determine the clinical impact of different drug resistance mutations at different concentrations. If minority variants are clinically important, use of more-sensitive assays might aid in the selection of potent ARV regimens with greatest chance of success. On the other hand, if minority variant drug resistance mutations have minimal clinical impact in ARV-naïve individuals, the detection of minority variants might lead care providers to prescribe complex first-line ARV regimens with a high pill burden and frequent dosing. Higher complexity of ARV regimens could reduce patient adherence and lead to a paradoxical increase in the prevalence of drug resistance. Given the uncertainty regarding the clinical impact of minority variant mutations and the fact that many people with these mutations still have excellent responses to therapy, prospective randomized trials that include cost-effectiveness analyses should be completed prior to the adoption of more-sensitive assays for clinical care.
